# Saliva

**Published:** 1887-05

**Authors:** C. Vaughn


### THE DENTAL REGISTER.

VOL. XLI.] MAY, 1887. [No. 5.

### COMMUNCATONS.

### Saliva.

BY PROF. C. VAUGHN.

(Read before the Michigan State Dental Society, March 23d, 1887.)

The composition of the saliva is probably of more interest to the dentist than to any one else. However, if the public at large knew something of the composition of this secretion it would be better for both dentist and patient. If people understood that tartar deposited on the gums is likely to do serious and even irreparable mischief, the dentist would be consulted more frequently and much expense as well as pain and disfigurement would be avoided. I tell my students in my lectures on Sanitary Science to visit some capable dentist once or twice a year, at least, and have the teeth thoroughly cleansed, and learn whether or not any other treatment of the oral cavity be necessary. With many, it will be found impossible to prevent altogether the deposit of tartar by the use of the tooth brush, and when unremoved it causes absorption of the gums, destroys the support of the teeth, and seriously interferes with their nutrition. This much, and even more, might be said in regard to tartar, and it is only one of the many constituents of the saliva which, when neglected, may produce harm. The mixed saliva is a turbid bluish white, and devoid of odor or taste. The normal reaction is alkaline and the alkalinity is increased during mastication, but during fasting the alkalinity decreases, and after prolonged fasting, the saliva may be faintly acid. In some diseased conditions the reaction may be acid. This is especially the case in certain forms of dyspepsia. The lodgment of particles of food between the teeth, and their retention and putrefaction there, lead to the formation of certain organic acids which not only destroy the alkalinity of the saliva, but irritate the mucous membrane of the mouth and lead to abnormal changes in its structure. In a mouth containing decomposing particles of food, certain forms are likely to be present, and these, or at least some of them, are to be regarded as the causes of disease.

For recent advances in our knowledge of the bacteriology of the mouth, we are especially indebted to Prof. Miller, of the Dental Department of the University of Berlin, and I may be pardoned for saying here with some pride that Prof. Miller is not a German, but an American, and a graduate of the U. of M. where I knew him as a classmate.

Among the most important minor organisms which may be found in the mouth are the following: spirochetae dentium, fine thread-like bodies from ten to thirty inches long, and with rapid movements. They are especially abundant in gingivitis. The leptothrix buccalis is a long, twisted, thread-like bacillus, which is stained blue by iodine and an acid. It was once supposed to be the cause of the dental caries, but this is now known to be an error. Its effect upon the mouth is not known. The leptothrix gigantia is very large and was found by Prof. Miller in the saliva of a dog suffering from pyorrhoea alveolaris. The Miller bacillus is comma shaped, is formed in the mouth and is supposed to be identical with the Tinkler-Prior bacillus. The Miller-Lewis bacillus is also comma shaped, but has not as yet been definitely studied. This is also called vioric buccalis. The bacillus of leprosy may also be found in the mouth, and a peculiar bacillus found in the mouth of a child which died at Lyssa from a new oral disease was discovered by Pasteur and is known as bacillus Lyssai. It was considered by Pasteur as the cause of the disease. Besides these, bacterum termo and other agents of putrefaction may be found in the mouth. The specific gravity of saliva varies from 1002 to 1009, the usual variation being from 1004 to 1006. Upon standing, saliva forms a greyish deposit which, by examination with the microscope, will be found to consist of pavement epithelium, mucous corpuscles, the debris of food and containing micro-organisms. Owing to the mucine which it contains, saliva is somewhat viscid; and can be drawn out into threads after it has been stirred for a few minutes with a glass rod. Owing to this viscidity it entangles and retains solid particles which it deposits in the mouth and thus aids in the formation of tartar. All other animal fluids probably decompose more rapidly than saliva. This is still another reason for keeping the mouth clean and free from deposits about and between the teeth. More than forty years ago Dr. Wright made some experiments which seemed to show that the saliva from healthy persons was poisonous to both vegetable and animal life: but his results are explained by the fact that he used putrid saliva.

The chief inorganic constituents of saliva are chlorides, sulphates, and phosphates of sodium, potassium, calcium, and magnesium. The putrid saliva contains calcium carbonate which, when the saliva is poured out from the duct, is held in solution by carbonic acid gas. In the mouth the gas escapes and the carbonate is deposited, thus aiding in the formation of tartar. Sulphuric acid combined with potassium is known to be a peculiar constituent of saliva. Why the salivary glands should elaborate this complex substance is not known. The chief organic constituents are albumen, mucine, and the spinal ferment ptyaline. An excess of mucine and albumen leads to a rapid decomposition and hastens putrefactive changes.

It is well known that certain abnormal substances, such as pus and bile, may be found in the saliva in diseased states. It is also well known that mercury, bromides, iodides, and other medicinal agents affect the salivary glands causing undue secretion and are poured out into the mouth with the saliva. It would not be desirable in a paper like this to introduce methods of analysis. That the dentist has excellent opportunities for securing specimens all know, and that much knowledge might be gained by making use of these opportunities is evident. A microscope, a few chemicals, a little time, which is often the most difficult to be obtained, with skill and perseverance furnish the desired equipment.

